# Cardiovascular imaging approach in pre and postoperative tetralogy of Fallot

**DOI:** 10.1186/s12872-018-0996-9

**Published:** 2019-01-07

**Authors:** Sotiria C. Apostolopoulou, Athanassios Manginas, Nikolaos L. Kelekis, Michel Noutsias

**Affiliations:** 1 Department of Pediatric Cardiology & Adult Congenital Heart Disease, Onassis Cardiac Surgery Center, 356 Syngrou Ave, 176 74 Athens, GR Greece; 2 Interventional Cardiology and Cardiology Department, Mediterraneo Hospital, Athens, Greece; 3 2nd Department of Radiology, General University Hospital “ATTIKON”, Medical School, National and Kapodistrian University of Athens, Athens, Greece; 4 Mid-German Heart Center, Department of Internal Medicine III (KIM-III), Division of Cardiology, Angiology and Intensive Medical Care, University Hospital Halle, Martin-Luther-University Halle, Ernst-Grube-Strasse 40, D-06120 Halle (Saale), Germany

**Keywords:** Angiocardiography, Cardiac magnetic resonance, Congenital heart disease, Echocardiography

## Abstract

Advances in the medical and surgical management of Tetralogy of Fallot have led to marked increase of the number and age of survivors. Imaging in patients with Tetralogy of Fallot plays a crucial role in the diagnosis and follow up, and essentially guides management and intervention in this entity. This study systematically reviews the imaging modalities used in patients with Tetralogy of Fallot in the evaluation of preoperative and postoperative anatomic and hemodynamic lesions, as well as disease progression in this diagnosis. Various invasive and noninvasive imaging modalities, most commonly echocardiography and cardiovascular magnetic resonance, computed tomography and angiocardiography provide the imaging information required for diagnosis, management and follow up in Tetralogy of Fallot. The choice of the appropriate imaging tool or their combination is guided by the clinical question, the patient’s clinical condition and contraindications as well as the strengths and weaknesses of each imaging modality. Tetralogy of Fallot is the most common complex congenital heart disease with long term survivors that need close follow up and complicated management, including multiple surgical and transcatheter interventions. Knowledge of the role and protocols of imaging in Tetralogy of Fallot is extremely important for the clinical as well as the imaging physician in order to optimize patients’ management and long-term prognosis.

## Background

Tetralogy of Fallot (TOF) represents 7–10% of congenital heart disease (CHD) occurring in 0.5/1000 live births and is the second most common form of complex CHD. Although frequently fatal without surgical intervention, advances in one or two-stage surgical repair in the recent era facilitates survival of the affected patients into adulthood with good quality of life, albeit with long-term complications and residual lesions [1]. The long-term survival and complexity of this entity creates the need for organized imaging approach, including multiple modalities [2], in order to optimize follow up, timing of reoperations and general wellbeing of these patients. Several diagnostic tools can be used, either alone or in combination, for imaging purposes in TOF, depending on the indications, the patient’s age and clinical condition, availability and local expertise, the cost of each tool as well as the possible need for intervention. This report aims to clarify the indications and role of each imaging modality in the diagnostic and therapeutic approach of preoperative and postoperative TOF.

## Anatomy and physiology in tetralogy of Fallot

**Fig. 1:**
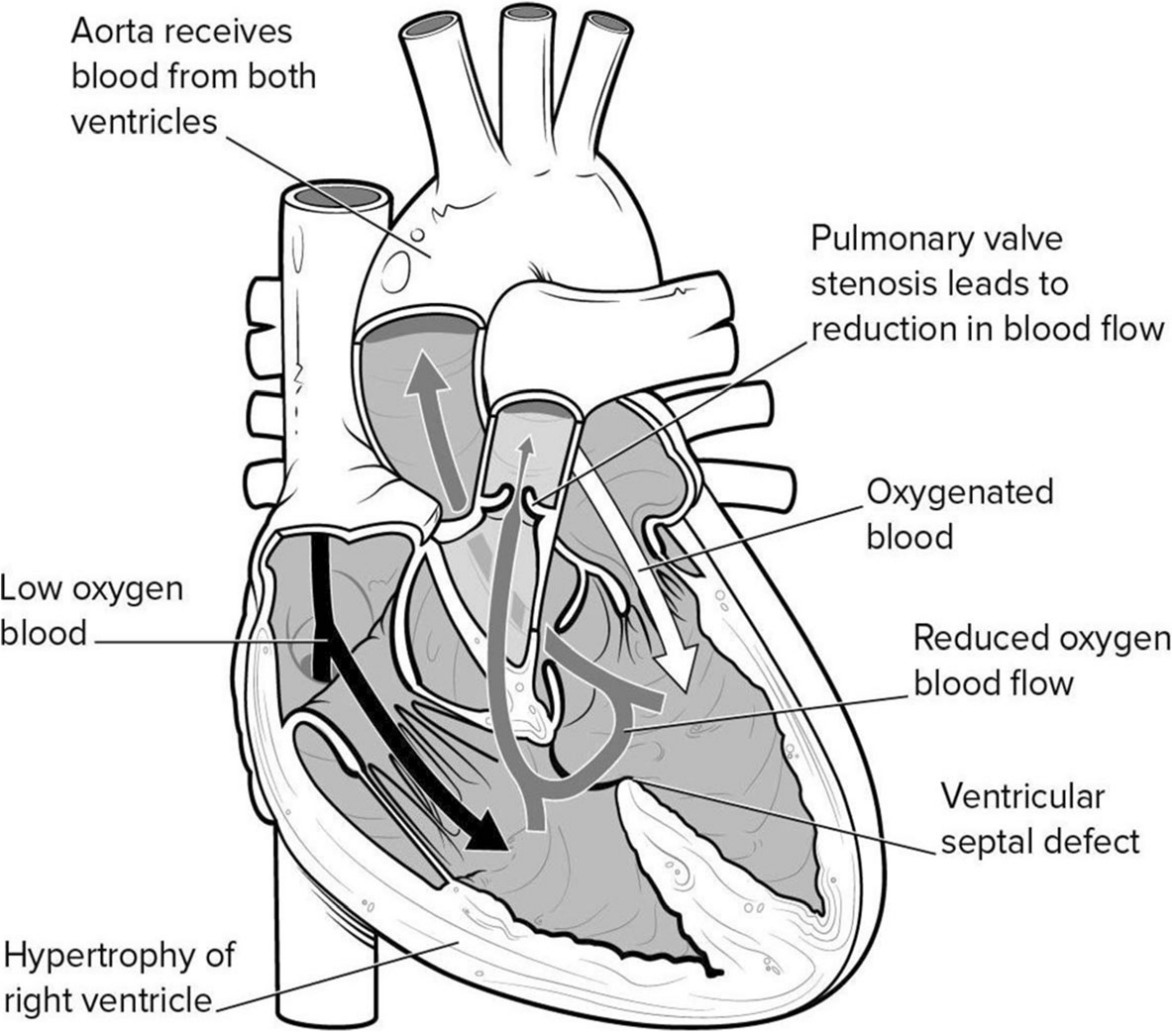
Diagram of the anatomy in Tetralogy of Fallot (Image reproduced with permission from Medscape Drugs & Diseases (https://emedicine.medscape.com/article/2035949-overview 2018)

Despite its name suggesting four anatomic defects, TOF is produced solely by anterior deviation during embryogenesis of the infundibular septum which separates the outflows of the two ventricles [3]. Such anterior deviation of the infundibular septum displaces it under the pulmonary valve creating the four elements of TOF: (a) subpulmonary obstruction resulting in (b) right ventricle (RV) hypertrophy, and (c) malalignment ventricular septal defect resulting in (d) apparent overriding of the aorta over both ventricles (Fig. 1).

The clinical picture in preoperative TOF evaluation depends on the degree of obstruction to pulmonary blood flow and is characterized by various grades of cyanosis and, more rarely, by heart failure when left to right shunt predominates. The degree of pulmonary blood flow determines the size and morphology of the pulmonary arteries, which are frequently hypoplastic, stenotic or inadequately arborized. Associated anomalies in TOF include atrial or additional ventricular septal defects, mirror-image right aortic arch without hemodynamic significance in 25% of subjects, tricuspid valve abnormalities, aortopulmonary collaterals, and anomalous origin and course of coronary arteries that may impact on surgical strategy [3].

Surgical palliation in TOF is achieved by creation of systemic to pulmonary artery shunts to improve cyanosis prior to repair. Surgical, usually transatrial/transpulmonary, repair in TOF is increasingly used primarily even in small infants, and consists of ventricular septal defect closure and relief of RV outflow tract obstruction by a combination of RV muscle resection, infudibulotomy and patch enlargement of the RV outflow tract and the pulmonary arteries when needed [4]. Attempts to preserve pulmonary valve function with valve sparing techniques to reduce pulmonary regurgitation may result in residual stenosis. Postoperatively, the clinical course is dependent on the degree of tricuspid and pulmonary regurgitation (PR), pulmonary obstruction and RV dilation, scarring and dysfunction, while long term complications include aortic root dilation, left ventricle (LV) dysfunction as well as conduction and rhythm disturbances [3].

## Construction and content

### Imaging tools in tetralogy of Fallot

The ideal imaging tool in TOF should be able to adequately assess all its structural and functional abnormalities both pre and postoperatively. Before operation, it is thus important to depict the size, morphology, arborization and possible stenoses of the pulmonary arteries, the infudibular obstruction, the presence of additional ventricular or atrial septal defects, the origin and proximal course of the coronary arteries, the source of extracardiac pulmonary blood flow and the size, morphology and function of the RV and the tricuspid valve. Additionally after repair, the imaging tools should evaluate RV volume and pressure overload due to tricuspid and pulmonary regurgitation or stenosis, RV and LV systolic and diastolic dysfunction, presence of postoperative scars, RV aneurysms and fibrosis, and associated anomalies such as aortic root dilation and aortic insufficiency. Coronary anatomy and coronary artery disease may need to be assessed in older patients with comorbidities such as hypertension and atherosclerosis.

No single diagnostic modality suffices for complete evaluation of TOF at any stage. All the diagnostic tools discussed below have their strengths and weaknesses (Table 1) and should be used alone or in combination according to the clinical question, the age, size and clinical condition of the patient, the availability, cost and radiation burden of each modality, as well as the need for sedation and the contraindications of each patient.

**Table 1:** Strengths and weaknesses of major imaging modalities in TOF

Characteristic	ECHO	CMR	CCT	CCA
RV size and function	++	++++	+++	+++
RV pressure	+++	+	+	++++
Severity of tricuspid/pulmonary stenosis	+++	++	–	++++
Severity of tricuspid insufficiency	+++	+++	–	+++
Severity of pulmonary insufficiency	++	++++	–	+++
Branch pulmonary arteries imaging	+	+++	+++	+++
Branch pulmonary arteries flows	–	+++	–	–
Aortopulmonary collaterals	–	+++	+++	+++
Imaging of left to right shunts	+++	+++	+	+++
Quantification of left to right shunts	+	++++	+	++++
LV size and function	+++	++++	+++	+++
Myocardial viability	+	++++	+	+
Aortic root imaging	+++	++++	++++	++++
Coronary origin and proximal course	++	+++	++++	++++
Coronary angiography	–	+	+++	++++
Sedation needed in young patients	++	++++	+++	+++
Availability and local expertise	++++	++	++	++
Ionizing radiation	–	–	++++	+++

*CCA* cardiac catheterization and angiocardiography, *CCT* cardiovascular computed tomography, *CMR* cardiac magnetic resonance, *ECHO* echocardiography, *LV* left ventricle, *RV* right ventricle

### Chest radiography and fluoroscopy

The chest radiograph is usually one of the first studies performed, especially when initially suspecting the diagnosis of TOF. It is inexpensive, readily available, and quickly provides information on gross cardiac and mediastinal size and configuration, situs of the aortic arch, abdomen and thorax, pulmonary vascularity, presence of calcifications or thoracic cage abnormalities and postoperative changes [5].

**Fig. 2:**
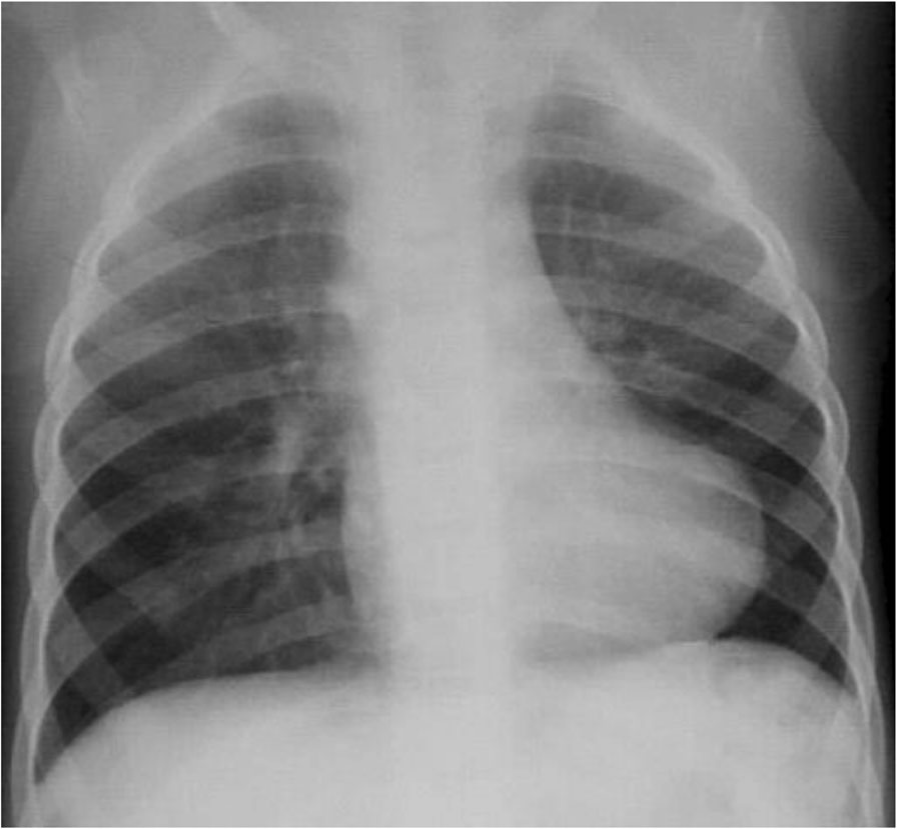
Chest radiograph of child with TOF showing the classical “boot-shaped heart” due to the narrow mediastinum and RV hypertrophy with decreased pulmonary vascular markings

Preoperatively in the neonatal period, the chest radiograph is mainly used to assess pulmonary vascularity and degree of pulmonary blood flow, while, as time evolves, the cardiac silhouette progresses to the classical “boot-shaped heart” explained by the narrowing of the mediastinum due to hypoplasia of the RV outflow tract and upward displacement of the RV apex due to its hypertrophy (Fig. 2). After repair, the chest radiograph may show cardiac enlargement due to PR or depict differential blood flow to various lung segments. Obviously, chest radiography alone is insufficient diagnostic tool and is used in conjunction with other modalities.

Fluoroscopy can be helpful in evaluation of postoperative abnormalities, such as paralysis of the hemidiaphragm, or in assessment of associated vascular anomalies compressing the trachea and esophagus using barium esophagram [6]. Fluoroscopy is quick and readily available and may still have a role if CMR is difficult to obtain, or in neonates where clinical condition or sedation requirements make a CMR investigation challenging.

## Echocardiography

### Overview of echocardiography

Transthoracic echocardiography is the cornerstone of imaging in TOF both pre and postoperatively, as it provides information on septal anatomy, RV and LV size and function, tricuspid and pulmonary stenosis and regurgitation, proximal pulmonary artery anatomy and stenosis, aorta size and insufficiency, and hemodynamic indicators such as estimated RV and pulmonary pressures. It is also widely available, portable, reproducible and safe [5], with widespread use and good level of expertise among clinicians. Moreover, transesophageal, 3D and tissue Doppler echocardiography can further aid in RV and valve morphology and function in TOF. Disadvantages of echocardiography include limited acoustic windows, especially in older and overweight patients, thoracic cage and postoperative abnormalities as well as the inability to provide reliable quantitative data on RV size and function, issues increasingly important in repaired TOF.

The scanning protocol in transthoracic echocardiography should include all the standard echocardiographic views in the subxiphoid, apical, parasternal and suprasternal windows in a combination of complete sweeps as well as multiple selected single planes. In order to avoid oversights, data acquisition should follow a standard protocol including the preferred order, the imaging tools recorded in each view and the recommended measurements [7] for a pediatric echocardiogram, while modified views may be needed, especially in complex TOF.

### Preoperative transthoracic echocardiographic assessment in TOF

Preoperatively, especially in the neonate, echocardiography can depict site and number of septal defects, degree of septal malalignment and resultant subpulmonary and pulmonary obstruction, anatomy of proximal pulmonary arteries, origin and proximal course of coronaries, presence without full delineation of aortopulmonary collaterals, and RV, tricuspid and pulmonary valve size and qualitative function. This information is usually adequate for planning surgical repair in the average patient, while further imaging is reserved for more complex cases, especially with hypoplastic or stenotic pulmonary arteries or need to visualize coronary course and aortopulmonary collaterals.

### Postoperative transthoracic echocardiographic assessment in TOF

**Fig. 3:**
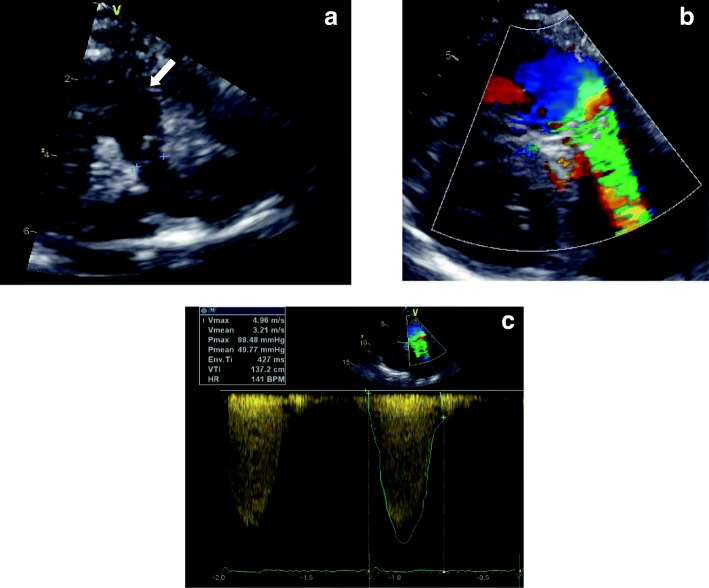
Echocardiography in postoperative TOF. **a** Parasternal short axis view of pulmonary valve (*crosses*), right ventricular outflow tract (*arrow*), main and branch pulmonary arteries. **b** Turbulence along stenotic pulmonary valve of an RV to pulmonary artery conduit without stenosis in the RV outflow tract. **c** Doppler tracing along the stenotic conduit indicates severe stenosis with maximal pressure gradient 98 mmHg

Postoperatively, echocardiography is very useful in evaluation of the RV outflow tract anatomy, size and dynamic or anatomic obstruction (Fig. 3), presence of aneurysms and the main and proximal pulmonary artery size, anatomy and residual stenosis. The degree of PR is evaluated usually in the parasternal views, assessing the diastolic flow reversal in the main or branch pulmonary arteries, the PR jet width, pressure half-time and index, that is the ratio of PR duration versus total diastole [8].

Several echocardiographic parameters have been shown to have reasonable correlation with CMR measured PR with variable specificity and sensitivity [9]. PR may be underestimated echocardiographically in the case of “free” or “wide open” PR or in elevated RV end diastolic pressures when the PR jet is laminar with low flow velocity [2].

Tricuspid regurgitation (TR) presence can be depicted using color and spectral Doppler in several views, while severity is assessed mostly qualitatively or measuring the vena contracta width [10]. Using the modified Bernoulli equation in the TR jet and the right atrial pressure according to the right atrial size and inferior vena cava respiratory variation, echocardiography can estimate RV pressure as well as pulmonary pressure in the absence of residual pulmonary obstruction [11].

RV size and function is usually evaluated qualitatively by echocardiography in several views, despite known poor interobserver agreement [12]. Objective RV assessment presents difficulties in imaging the entire RV due to its crescentic, nongeometric shape and its retrosternal position. RV end diastolic cross-sectional area measured from an RV focused apical view correlated with CMR derived RV volume [13], while RV fractional area change (RV % shortening) combined with RV outflow tract fractional shortening has reasonable correlation with RV ejection fraction by CMR [14]. Echocardiographic 3D reconstruction significantly improves correlation of measured RV volumes with CMR data, although it presents several technical issues in dilated RVs and tends to underestimate RV volumes [15].

Indirect measures of RV function such as rate of pressure rise in the RV (dP/dt), myocardial acceleration during isovolumetric contraction and the myocardial performance index (Tei index) have not been validated in TOF [2]. Tricuspid annular plane systolic excursion (TAPSE), measuring the distance of systolic movement of the lateral tricuspid valve annulus in the apical four-chamber view, is generally considered to reflect global RV function. TAPSE is not a reliable index of RV function in TOF since correlation with CMR measured RV volumes and function is weak at best [16]. Tissue Doppler parameters of the tricuspid annulus and the RV free wall show reasonable correlation with CMR data in TOF [17], while myocardial deformation indices such as strain and strain rate are abnormal in repaired TOF for both the RV and the LV as a result of ventricular-ventricular interaction [18]. Diastolic RV dysfunction can be assessed using a combination of transtricuspid Doppler tracing, late diastolic antegrade flow in the pulmonary artery, right atrial dilatation, hepatic venous flow reversal, and inferior vena cava respiratory changes, but values for TOF have not been ascertained [2].

Echocardiography evaluation in TOF has shown abnormalities in right atrial size and emptying [19], as well as LV size and function [20]. It can also assess the presence of residual shunts and aortic dilation, a common long term complication of repaired TOF. Further information on most previously described echocardiographic parameters is needed in TOF before they can be used in clinical practice for patient follow up and prognosis.

### Other echocardiographic techniques in TOF

Transesophageal echocardiography (TEE) in the hands of a skilled operator in CHD plays an important role in preoperative evaluation of the tricuspid valve and septal defects, in intraoperative assessment of the repair and postoperative imaging of vegetations, RV to pulmonary artery conduits, implanted pulmonary valves, and functional assessment of ventricles and valves, especially in the case of poor acoustic windows in transthoracic studies. Stress echocardiography, either with exercise or dobutamine, may provide useful information on biventricular function in TOF, especially in a population with increasing age that may develop coronary artery disease [21].

## Cardiac magnetic resonance (CMR)

### Overview of CMR

CMR is invaluable for TOF imaging preoperatively and, most importantly postoperatively. Traditional “black-blood” techniques, such as turbo or fast spin-echo and double inversion recovery fast spin echo sequences can demonstrate static images of cardiac and pericardiac anatomy with high spatial resolution and contrast between elements [22]. “Bright-blood” techniques, such as cine steady-state free precession or gradient echo pulse sequences can depict flow jets in septal defects, valvar stenosis or insufficiency, or stenotic lesions. “Bright-blood” techniques can also be used for volumetric coverage to measure right and left ventricular volumes, ejection fractions, and myocardial mass with high reproducibility [23]. Newer techniques have shortened acquisition times so that bright-blood imaging can be performed during a short breath hold. High-resolution cardiac-gated MR angiography of the heart and great vessels can now be obtained using free-breathing techniques, such as blood-pool contrast enhancement with or without gadolinium contrast, using a high native-contrast steady-state free precession sequences and allowing for multiplanar reformats and 3-D imaging of the heart and coronaries. In addition, phase-contrast techniques show direction and permit quantification of blood flow, allowing thus identification of intracardiac or extracardiac shunts and calculation of pulmonary to systemic blood flow ratio (Qp/Qs), pressure gradients across stenotic lesions, regurgitant fractions across incompetent valves, and aortopulmonary collateral flow [5]. Late gadolinium enhancement (LGE) technique in repaired TOF assesses myocardial perfusion, ischemia and scar tissue that may be associated with impaired mechanics and adverse outcome [24].

CMR has no ionizing radiation, can be performed irrespective of patient’s body habitus or imaging windows. Unlike echocardiography that is mostly used qualitatively, CMR provides objective, accurate and reproducible quantitative measurements of RV size and function, valve regurgitation, pulmonary and systemic flows, differential pulmonary artery flow and myocardial scar tissue detection [2], all parameters being extremely important in long-term sequential follow up of TOF. Absolute or relative contraindications include allergy to gadolinium, presence on non-CMR compatible pacemakers and defibrillators, renal insufficiency, claustrophobia, and need for sedation in younger patients. CMR may not be ideal in depiction of small septal defects, calcifications or metallic artifacts common in postoperative TOF, especially in conduits and intravascular stents. It also has elevated cost and requires significant medical and technological personnel expertise both for image acquisition and postprocessing.

CMR is less frequently used in preoperative TOF in young patients, where echocardiography offers good imaging and is usually sufficient for surgical planning. Preoperatively, CMR is reserved for complex anatomy, such as pulmonary atresia for better delineation of the frequently hypoplastic pulmonary branches and the aortopulmonary collaterals. Postoperatively, CMR is rarely needed in preadolescent patients unless ventricular dysfunction or right heart failure necessitates further workup and possible intervention. The role of CMR becomes crucial in older postoperative TOF patients, where serial evaluations guide management and planning of surgical or interventional pulmonary valve replacement according to well validated, heavily relying on CMR criteria.

### CMR assessment in TOF

The CMR protocol in repaired TOF includes cine evaluation of valve function and anatomy of the RV, the RV outflow tract, the LV and the ventricular septum. Measurement of ventricular volumes and of mass is then performed with special attention to complete chamber coverage, especially in the basal sections as well as correct determination of end-diastole and end-systole for each ventricle given the usual RV conduction delay [25]. Imaging for volume measurements should be performed in multiple views, including RV and LV two-chamber and four-chamber views and RV and LV outflow tract long-axis and short-axis views. Using the measured end-diastolic and end-systolic ventricular volumes, stroke volumes and ejection fractions are derived for each ventricle, while ventricular mass is calculated by subtracting endocardial from epicardial volume for the RV and the LV respectively, knowing that systolic measurements show more reproducibility [26]. CMR parameters of RV size, function and hypertrophy have been identified as predictors of death and ventricular tachycardia in repaired TOF [27].

**Fig. 4:**
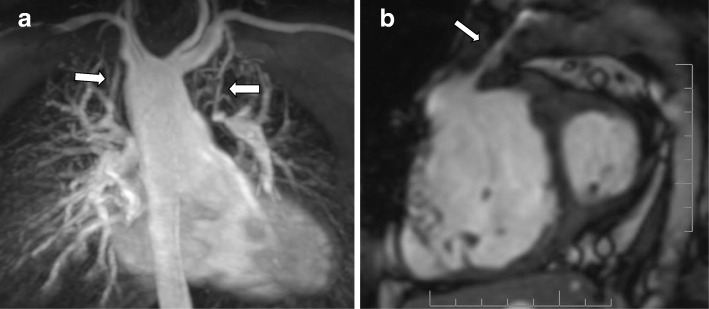
CMR in TOF. **a** CMR angiography showing mirror-image right aortic arch, anatomy of the pulmonary branches and supply of both pulmonary arteries by bilateral modified Blalock-Taussig shunts (*arrows*), without connection of the left pulmonary to the main pulmonary artery. **b** CMR white-blood cine frame in the short axis plane shows a dilated, hypertrophic RV compressing the LV and the very stenotic, threadlike conduit from the RV to the pulmonary artery (*arrow*)

Magnetic resonance (MR) angiography with gadolinium contrast is then used for depiction of pulmonary arteries, branches and collaterals (Fig. 4a) as well as the RV outflow tract, possible aneurysms or complex geometry, stenotic or dyskinetic regions and conduits to the pulmonary arteries (Fig. 4b), information very useful in surgical and interventional planning, especially in pulmonary valve replacement [28].

**Fig. 5:**
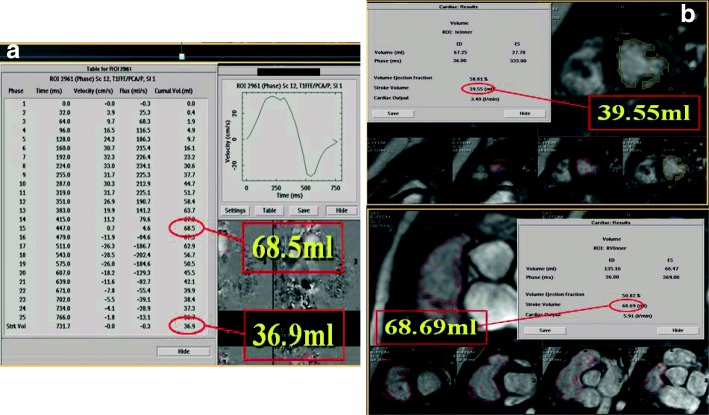
CMR flow and volume measurements in repaired TOF used to evaluate PR. **a** Flow measurements in the main pulmonary artery indicate increased antegrade flow in systole (68.5 ml) with significant retrograde flow in diastole (36.9 ml) also depicted in the upper left flow diagram, calculating a PR fraction of 46%. **b** Volume measurements of the RV and LV show significantly increased RV stroke volume compared to the LV due to the severe PR. Both flow and planimetry derived RV forward volumes during systole coincide due to absence of significant tricuspid regurgitation

Subsequently, flow measurements are performed in planes perpendicular to the blood flow to quantify pulmonary or other valve regurgitation, differential pulmonary artery flow and pulmonary and systemic flow (Fig. 5a). Fractional pulmonary regurgitation calculated as the ratio of retrograde to antegrade flow as well as the absolute retrograde volume through the pulmonary valve area is calculated [29]. In the absence of other significant valve lesions or left to right shunt the degree of PR is also reflected by the difference in RV and LV stroke volumes (Fig. 5b).

**Fig. 6:**
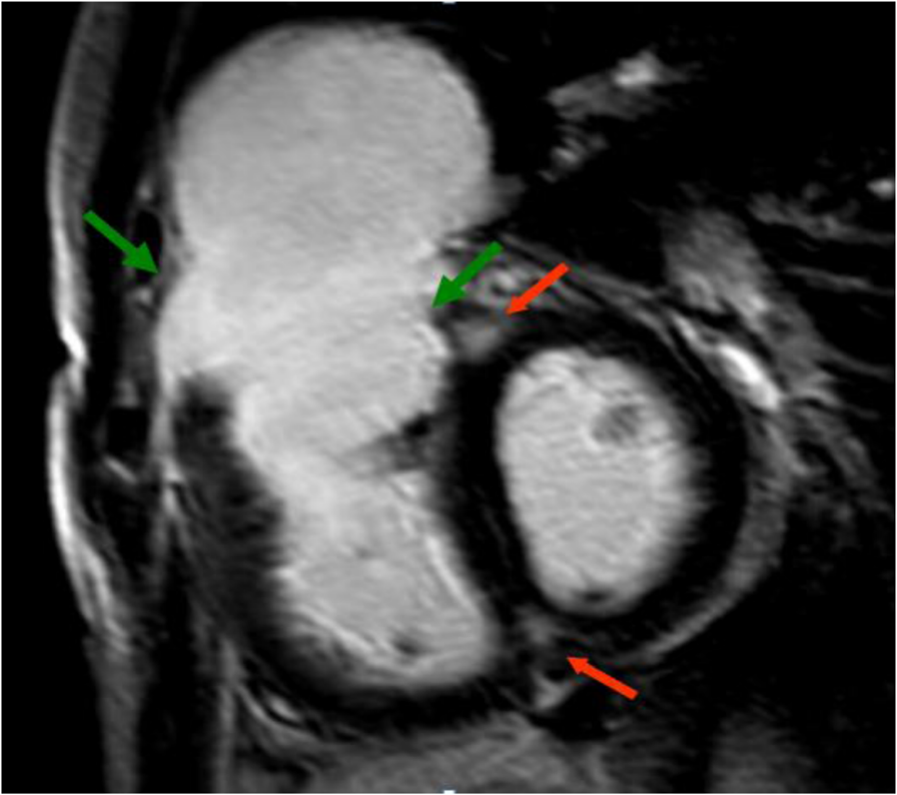
CMR in repaired TOF showing late gadolinium enhancement in the RV outflow tract (*green arrows*). Of note, late gadolinium enhancement at the hinge points of the ventricular wall to the septum (*red arrows*) is common and nonspecific in TOF

Lastly, late gadolinium enhancement (LGE) 10 to 20 min after contrast injection evaluated in multiple planes assesses myocardial viability and presence of scar tissue in the myocardium (Fig. 6). Myocardial enhancement at the hinge points of the ventricular wall to the septum is very common in repaired TOF but if present in other regions may be related to decreased exercise tolerance and ventricular dysfunction [30].

CMR objectively evaluates right atrial size, function and emptying influenced by TR and RV function, depicts arch sidedness and anomalies and offers excellent visualization and serial measurements of the aortic root and ascending aorta that are frequently dilated in TOF. CMR also assesses size, global and regional dysfunction of the LV, present in the late stages of repaired TOF due to ventricular-ventricular interaction, which has been associated with adverse outcome, death and ventricular tachycardia [31].

## Cardiovascular computed tomography (CCT)

### Overview of CCT

Multidetector CCT in the current era offers excellent spatial and reasonable temporal resolution producing static and cine imaging as well as 3D reconstructions and can be very helpful in TOF evaluation. Cardiac gating is not always necessary for extracardiac structures but is of the utmost importance for intracardiac or coronary imaging and heart rate control with β-blockers is advocated for optimal acquisition, when not contraindicated.

Compared to CMR, CCT provides superior delineation of small vessels, aortopulmonary collaterals and pulmonary arteries, is less affected by stainless steel artifacts and has shorter acquisition times reducing the need for sedation, all qualities that may prove important in selected TOF patients [5]. Coronary computed tomography angiography (CCTA) has been shown to provide high resolution and quality depiction of the origin and course of coronary arteries [32], an important issue for surgical planning in preoperative TOF, as well as in older TOF patients with possible coronary abnormalities or proximity of the RV outflow tract to coronary vessels that may get obstructed during percutaneous pulmonary valve implantation [33]. CCT imaging does not interfere with pacemakers and defibrillators, even the older, non-compatible with CMR models.

Disadvantages of CCT include its inability to provide reliable flow rate or velocity data and the use of ionizing radiation with its possible risks. Recent advances and experience with variable pitch and low kilovolt techniques tailored to the pediatric population have led to a tenfold reduction in radiation dose and permit cardiac gated CCT in children with low doses in the order of 1 mSv [34]. Newer techniques reducing radiation dose such as prospective triggering, adaptive statistical iterative reconstruction, and high-pitch spiral acquisition are not widely usable yet as they depend on heart rate, rhythm, body habitus and type of scanner but may further decrease the dosage in cardiac and coronary CT angiography [5]. Use of contrast media during CCT may aggravate preexisting renal dysfunction but is associated with overall few adverse events with the low osmolality agents [35].

### CCT assessment in TOF

**Fig. 7:**
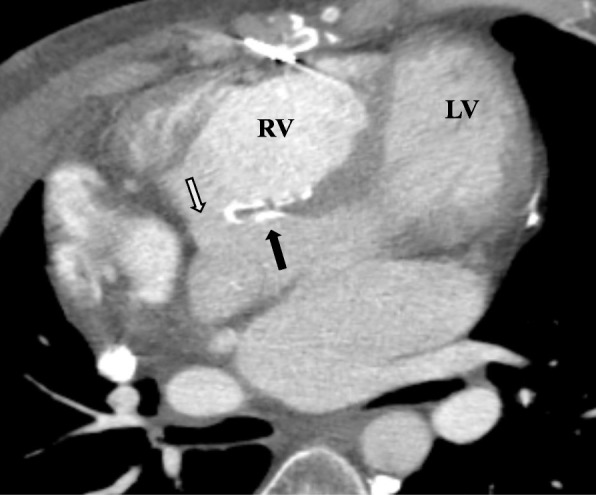
CCT in postoperative TOF showing dilated RV with bowing of the ventricular septum towards the LV, the surgical ventricular patch (*black arrow*) and a significant residual ventricular septal defect (*white arrow*). LV, left ventricle; RV, right ventricle

The protocol for CCT assessment in TOF includes visualization of pulmonary artery anatomy, size and arborization, especially in cases with prior aortopulmonary shunts [36], as well as measurement of the lumen and wall thickness of the ascending aorta in older patients and comparison with normal values [37], since progressive root dilation is common in TOF as in other conotruncal diseases. Biventricular end-diastolic and end-systolic volumes can be measured by retrospective reconstruction of the electrocardiogram (ECG) gated acquired data set, from which LV and RV ejection fractions, stroke volumes and cardiac output can be calculated with reasonable accuracy compared to the CMR gold standard if the RV cavity is adequately opacified [38]. CCT demonstrates intracardiac anatomy such as ventricular septal defects, taking into account its relative deficiency in atrial septum and membranous ventricular septum visualization (Fig. 7). Finally, it is extremely helpful in depicting the origin and course of coronaries [32], as well as RV to pulmonary artery conduits and their relation to surrounding structures, thus enabling correct planning of surgical or transcatheter interventions.

Despite the good imaging quality of CCT in TOF, it should be reserved only for patients with absolute contraindications to CMR, given its ionizing radiation, the young age of the patients and the need for serial imaging throughout their lifespan.

### 3D printing

3D printed reconstruction using high resolution CT [39] or blood-pool contrast enhanced MR [40] data sets have been reported in complex CHD and TOF, in order to enhance understanding of the intracardiac and extracardiac anatomy, especially during planning for surgery or transcatheter intervention. Although this technique is costly, time consuming and requires significant operator experience, it is anticipated that such reconstructions will be increasingly used in the future as technology progresses, especially in complicated cases.

### Nuclear imaging

Traditionally, nuclear scintigraphy has been used in TOF for assessment of RV and LV size, function and myocardial viability at rest and during exercise, quantification of intracardiac shunts, and differential pulmonary perfusion between right and left lung [5]. Most of these indications are better served with CMR in the current era without the ionizing radiation risks, but with probably increased sedation requirements in small children compared to nuclear imaging. Still, nuclear scintigraphy using ^99m^Tc-labeled macroaggregates of albumin embolizing at the capillary level provides an accurate picture even in patients < 10 years of age or very small pulmonary arteries, except in cases of multiple sources of pulmonary blood flow [2]. Nuclear imaging retains its role in the assessment of differential lung perfusion, especially in the presence of left pulmonary artery stenosis and PR [41]. It also permits evaluation of pulmonary ventilation as well as ventilation perfusion mismatch [42], frequently present in repaired TOF due to thoracic cage and postoperative abnormalities. For the remainder of indications, nuclear imaging is used very sparingly and is reserved for patients with clear contraindications to CMR and cardiac CT [2].

## Cardiac catheterization and angiocardiography

### Overview of CCA

Cardiac catheterization and angiocardiography (CCA) has been the cornerstone for the diagnosis and management of CHD over the past 50 years, albeit increasingly replaced by the noninvasive modalities developed the last 25 years, initially echocardiography and, more recently, CMR. CCA provides invasive pressure and oximetry data in the pulmonary and systemic circulations, evaluates stenoses, direction and volume of shunts between the two circulations, pulmonary and systemic cardiac outputs by thermodilution or the Fick method, as well as pulmonary and systemic vascular resistances. Morphologic imaging is achieved usually by biplane cine angiograms in angulated views, using iodinated contrast material and ionizing radiation. Although invasive, CCA is relatively safe with few risks such as hematomas, arterial and venous injuries, renal function deterioration, contrast reactions, radiation exposure, and very low but definite mortality [5]. With the advent and optimization of noninvasive tools, CCA is reserved for delineation of complex CHD anatomy, such as TOF with pulmonary arterial tree abnormalities or prior aortopulmonary shunts, or during evaluation for possible transcatheter interventions, which are increasingly common in this population [43]. Still, CCA remains the gold standard for evaluation of intracardiac pressures, peripheral pulmonary arteries and coronaries and may well be used increasingly in this population with advancing age and possible coronary artery disease [2].

### CCA assessment in TOF

**Fig. 8:**
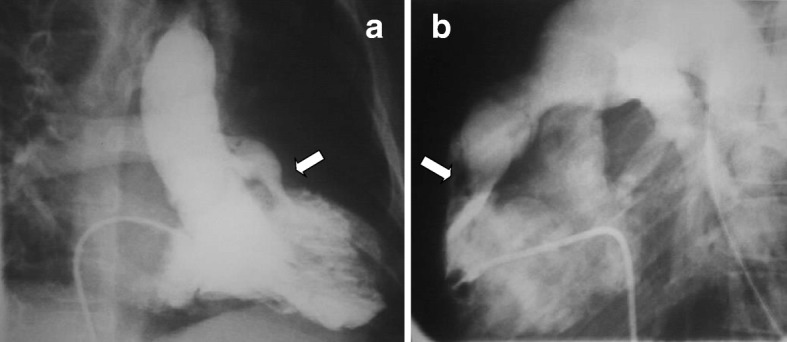
CCA in unrepaired TOF. **a** Right anterior oblique projection and **b** lateral projection of RV ventriculogram showing the hypertrophic RV ejecting most of the contrast to the overriding aorta, as well as the severe RV outflow tract obstruction (*arrow*) below the stenotic pulmonary valve

The protocol for CCA assessment in preoperative TOF (Fig. 8) depicts the ventricular septal defects, the subpulmonary and pulmonary stenosis, the branch pulmonary arteries, RV size and function and aortopulmonary collaterals that may need transcatheter occlusion if there is dual blood supply of a lung segment.

**Fig. 9:**
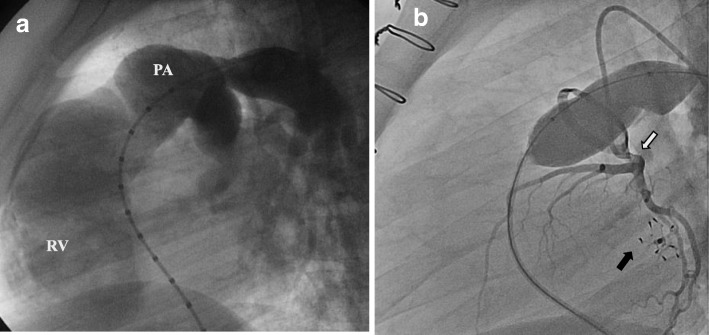
CCA in repaired TOF. **a** Lateral projection of injection in the right pulmonary artery showing severe PR with opacification of the whole RV cavity. **b** Left coronary angiography (*white arrow*) simultaneously with balloon interrogation in the bioprosthetic pulmonary valve to delineate the coronary origin prior to percutaneous pulmonary valve implantation. Note the atrial septal defect occluder (*black arrow*). PA, pulmonary artery; RV, right ventricle

In repaired TOF CCA evaluates, depending on the indication, the degree of PR, RV size and function qualitatively, the RV outflow tract and the pulmonary arteries and branches using multiple biplane angled views (Fig. 9a). Aortic root, arch and descending aorta injections assess aorta size, aortic valve insufficiency, and aortopulmonary collaterals, possibly amenable to transcatheter occlusion if indicated. Depiction of coronary origin and, if need be, selective coronary angiography during balloon interrogation of the RV outflow tract (Fig. 9b) is of paramount importance to exclude coronary compression during stenting of conduits or pulmonary valve implantation [33].

Rotational angiography with 3D reconstruction is a relatively new technique that can be very helpful in diagnostic and interventional CCA in TOF, especially in complex RV outflow tract anatomy and proximal pulmonary artery stenosis [44]. Data are acquired with rapid pacing and breath holding, minimizing radiation exposure, and the reconstructed 3D image can be overlaid on the active fluoroscopy, thus facilitating catheter manipulations and the choice of optimal camera angles [45].

## Utility and discussion

### Multimodality imaging in TOF

As becomes obvious from the strengths and weaknesses of each imaging tool (Table 1), no single imaging modality is sufficient for complete diagnosis and follow up in TOF [2]. Patients are mostly followed with yearly echocardiography in conjunction with physical examination, as well as periodic arrhythmia monitoring and cardiopulmonary exercise testing. All imaging modalities previously described, with the critical help from CMR, are used for establishing indications for surgical or transcatheter pulmonary valve replacement to alleviate the detrimental effect of PR on RV dilation and function. Indications for pulmonary valve replacement include severe diastolic and systolic RV dilation, decreased RV and LV ejection fraction, large RV outflow tract aneurysms, prolonged QRS or tachyarrhythmias, severe RV outflow tract or pulmonary branch stenosis, severe tricuspid or aortic insufficiency and significant residual septal defects [46].

Echocardiography represents the main imaging tool used at least on a yearly basis because it provides good functional assessment of the RV, pulmonary and tricuspid valves with low cost, wide availability and absence of contraindications or ionizing radiation. CMR, CCT and CCA are not routinely used before adolescence unless specific issues arise, and are then performed under sedation or general anesthesia. CMR is initially recommended in early adolescence and repeated approximately every 3 years to objectively assess RV size and function and PR. CMR is performed yearly in case of progressive RV dilation and dysfunction or significant right heart failure symptoms. CCT or CCA is reserved for patients with contraindications to CMR, in case of interventions, or need for coronary angiography. Lung perfusion scan is performed in case of considerable pulmonary branch stenosis, where significant decrease in ipsilateral lung perfusion is suspected and may warrant intervention.

Factors that should influence choice of imaging tool and adherence to the previously described protocol include availability and cost of each modality, local expertise and training, use of ionizing radiation and its potential risks, need for sedation in young patients, possible contraindications to a specific modality and, above all, the patient’s clinical condition and the parameters that need imaging for optimal diagnosis and follow up. These issues become increasingly important as we lack concrete data on the rate and predictors of disease progression in TOF, while economic factors greatly affect allocation of funds and resources in our current environment.

## Conclusions

Imaging in pre and postoperative TOF relies on multiple imaging tools used with variable frequency depending on patient’s age, clinical question, availability, cost and operator’s expertise. Further research is needed on the factors influencing disease progression in TOF concerning PR, RV dilation and dysfunction and aortic dilation in order to optimize management and follow up as well as timing of interventions in these patients.

## Abbreviations

CCA: Cardiac catheterization and angiocardiography
CCT: Cardiovascular computed tomography
CHD: Congenital heart disease
CMR: Cardiac magnetic resonance
CT: Computed tomography
ECG: Electrocardiogram
LGE: Late gadolinium enhancement
LV: Left ventricle
MR: Magnetic resonance
PR: Pulmonary regurgitation
RV: Right ventricle
TAPSE: Tricuspid annular plane systolic excursion
TOF: Tetralogy of Fallot
TR: Tricuspid regurgitation
